# Dual harm among patients in the Ontario forensic mental health system

**DOI:** 10.1371/journal.pone.0316364

**Published:** 2024-12-30

**Authors:** Mark Mohan Kaggwa, Bailea Erb, Sébastien Prat, Arianna Davids, John Bradford, Gary Andrew Chaimowitz, Andrew Toyin Olagunju

**Affiliations:** 1 Department of Psychiatry and Behavioral Sciences, McMaster University/St Joseph’s Healthcare Hamilton, Hamilton, Ontario, Canada; 2 Forensic Psychiatry Program, St Joseph’s Healthcare Hamilton, Hamilton, Ontario, Canada; 3 Department of Psychology, University of Waterloo, Ontario, Canada; 4 Michael G DeGroote School of Medicine, McMaster University, Hamilton, Ontario, Canada; 5 Division of Forensic Psychiatry, University of Ottawa, Ontario, Canada; 6 Department of Psychiatry and Behavioral Sciences, University of Oklahoma College of Medicine, Oklahoma City, OK, United States of America; 7 Discipline of Psychiatry, The University of Adelaide, Adelaide, SA, Australia; University of Toronto, CANADA

## Abstract

**Background:**

Dual harm involves the unfortunate experience of harm to self and others/objects. Safeguarding individuals in forensic psychiatric settings against all forms of harm to self and others is sacrosanct. While understanding dual harm is crucial in the care and rehabilitation of patients in forensic psychiatric settings, only a few studies have explored this phenomenon. This study examined dual harm and its associated clinical and sociodemographic factors among forensic patients in Ontario, Canada.

**Methods:**

In this retrospective study, we used data from the Ontario Review Board (ORB) (n = 1240; mean age 42.54±3.32 years, and 85.73% male). We defined dual harm as the co-occurrence of self-harming behaviour in the last 12 months and violent behaviour towards others or objects (such as verbal, physical, or sexual aggression). We analysed the data in relation to clinical and sociodemographic factors.

**Results:**

Of 1240 patients, 43 (3.55%) had engaged in dual harm. Most of them had engaged in dual harm related to verbal aggression (3.15%), followed by dual harm related to aggression towards objects (2.97%), dual harm related to aggression towards others (2.73%), and dual harm related to sexual aggression was the rarest (1.32%). Only 12 patients had engaged in all types of dual harm. Having a previous history of dual harm and a diagnosis of a neurodevelopmental disorder increased the chance of perpetrating/engaging in dual harm. However, increasing age and a higher education decreased the chance of dual harm. These factors were similar for different types of dual harm, except for dual harm related to sexual aggression, which was the only subtype associated with having a personality disorder diagnosis. Again, the experience of violence increased the chance of self-harm.

**Conclusion:**

Dual harm is present among forensic patients in Ontario, and self-harm is prevalent among individuals with various forms of aggression, especially when the violence was perpetrated towards objects. Strategies to manage the risk of self-harm among aggressive patients should be put in place to mitigate dual harm and the associated complications, especially among individuals with neurodevelopmental disorders.

## Introduction

### Overview of dual harm

Self-harm and violence are serious risky behaviours that can result in life-threatening or fatal outcomes. Both self-harming behaviors and violence are prevalent among individuals involved in the forensic psychiatric system [1–3]. Evidence from previous studies has shown a relationship between self-harming behaviors and violence, such that individuals who have displayed one form of harm are considered ‘at risk’ for exhibiting the other [3–6]. Consequently, displaying violence to both self and others/objects is referred to as dual harm [7]. The co-occurrence of self-harming behaviours and violence towards others (dual harm) can present unique issues for risk management and patient safety, necessitating a better understanding of the extent, nature, and source of dual harm among ‘at risk’ groups [5]. To this end, there is a growing body of literature on the clinical, policy, and administrative ramifications of dual harm behaviours in individuals in the criminal justice system and the general population [5]. While previous studies have highlighted the importance of understanding dual harm in forensic psychiatric populations [8], no recent study has reported this phenomenon and described the unique characteristics of patients within the forensic psychiatric system with dual harm. This study aims to address this gap and extend current literature on dual harm among the forensic psychiatric population. An overview of relevant literature in subheadings is included below to provide appropriate background for the study.

### Prevalence and incidence of dual harm

The prevalence of dual harm from previous studies ranged between 7.5% among forensic patients with a history of aggressive behaviors in China [9] and 19% among patients admitted to a forensic hospital in Sweden [10]. Among individuals in prisons (a comparable population to forensic psychiatric patients), the prevalence ranged between 5% and 28.13% [11, 12]. Despite some studies reporting a higher prevalence of dual harm among individuals in prisons, these prevalence rates are comparable with those obtained from forensic populations [9–14]. Dual harm has also been reported in other populations, including patients in general psychiatry settings, university students, children and adolescents, and individuals experiencing homelessness, among others [3]. While dual harm has been studied extensively in psychiatry, only a few studies have reported dual harm among forensic patients [9, 10]. In addition, the available research from Sweden, conducted over two decades ago, used a small sample [10]. Therefore, the current study is an important addition to the extant literature, reporting the prevalence of dual harm using a large sample of individuals in the forensic system in Ontario, Canada.

### The dual harm phenomena and related theories

It is considered dual harm when individuals engage in both self-harming behaviors and aggression towards others/objects [7, 8]. Several models have explained the theoretical underpinnings related to this dual relationship. Shafti et al. (2021) explained dual harm using the cognitive-emotional model [7]. This model incorporates the (i) general aggression model (based on a social cognitive theory that divides the explanatory factors of aggression into distal versus proximal factors) [15], (ii) diathesis-stress model (i.e., proposed that adverse childhood events interact with biological factors (e.g., genetics) to develop certain personality traits and cognitive styles that lead to impairment of self-regulation and decision making) [16], and (iii) emotional dysregulation theories that proposed four underpinning defects, including (the absence of “(a) awareness and understanding of emotions, (b) acceptance of emotions, (c) ability to control impulsive behaviours and behave in accordance with desired goals when experiencing negative emotions, and (d) ability to use situationally appropriate emotional regulation strategies flexibly to modulate emotional responses to meet individual goals and situational demands”) [17]. Central to these theories is the postulation that emotional dysregulation may lead to the use of self-harm and/or violence to relieve emotional distress; however, this may also lead to a positive feedback mechanism that will reinforce maladaptive behaviors in a classical conditioning format [7]. The interpersonal theory of suicide can also explain self-harm behaviors that follow acts of violence. This theory proposes that burdensome activities such as aggression towards others/objects will increase an individual’s desire or likelihood to engage in self-harming behaviours [18].

Furthermore, other individuals may engage in self-harming behaviors following aggressive incidents to seek out help or create boundaries for themselves from others [19]. Among female patients in a forensic psychiatric setting, dual harm was reported to be a coping strategy towards various physical and psychological stressors [20]. Another theory proposed by Slade et al. [20] implicated dysfunctional environments during childhood as a major determinant of whether an individual will engage in self-harm or dual harm. They theorized that damaging childhood environments can reinforce trauma and emotion-based distress later on in life, eliciting an individual’s “need” to dual harm [21].

### Factors associated with dual harm

Individuals with self-harming behaviours or suicidal ideations are reported to have a higher risk of being involved in aggressive incidences than those without self-harming behaviours [22–24]. Individuals who have experienced adverse childhood events, including child abuse and parental death, are at a higher risk of engaging in dual harm [9]. Trait impulsivity has been shown to be a significant predictor of dual harm, specifically in individuals with borderline personality disorder [25]. When it comes to psychiatric diagnoses, mood disorders have been associated with a higher risk of engaging in dual harm [9]. Furthermore, individuals with high levels of anxiety and depressive symptoms and those who developed psychosis before the age of 18 years were at a higher likelihood of engaging in dual harm [21]. Despite the highlighted literature describing factors related to dual harm, observational studies exploring this aspect of dual harm among patients within the forensic psychiatric systems are limited.

### Justification for the present study

Several ramifications of dual harm with notable clinical significance have been documented. For example, previous research has reported longer stays in the forensic system and lower participation in behavioural programs if individuals engage in dual harm [8]. Consequently, a better understanding of the factors associated with dual harm among forensic patients is important in addressing risk and reducing complications related to self-harm and or violence, such as suicide, infection, harm to others, and objects [8]. Again, an adequate estimate of the burden of dual harm in forensic settings using a population-based dataset (ORB data for all forensic programs in a Canadian province) can help inform the allocation of resources to guide the development of management strategies and stem the risk. In view of the aforementioned literature and considering the various explanatory theories suggested for the relationship between violence and self-harming behaviour (dual harm), the current study aims to:

1. Estimate the prevalence of dual harm (i.e., the co-occurrence of self-harm with any type of violence or aggression) among patients in the forensic psychiatric system in Ontario.
2. Determine the relationship of dual harm with clinical and sociodemographic characteristics factors among patients in the forensic psychiatric system in Ontario.
3. Determine the strength of the relationships between different aspects of aggression or violence and self-harm among patients in the forensic psychiatric system in Ontario.

## Methods

### Study design and population

This retrospective study analysed data involving patients under the Ontario Review Board (ORB) during the reporting years of 2014 and 2015. The present study was approved by the Hamilton Integrated Research Ethics Board (HiREB) [Approval reference: #15564]. HiREB waived the need for informed consent. The authors did not have access to information that could identify individual participants during or after data collection. Following ethical approval, the authors accessed the database on 15 December 2022.

The database was created to provide a population-based snapshot and perspective on individuals in 13 forensic psychiatric programs across Ontario for the abovementioned years [26]. To answer the study objective, the present study used data on violence towards others or objects, self-harming behaviours, socio-demographics, and clinical characteristics.

### Study variables

#### Past 12-month self-harming behaviours

Self-harming behaviour in the past 12 months was captured with *Yes* for presence and *No* for absence. This involved intentional self-harm and suicide attempts, but not unintentional self-harm (for example, accidentally falling and injuring oneself).

#### Violence history

This variable captured any incidents of aggression or violent activity prior to or during the reporting year. It was reported as *Yes* (presence of at least one incident) or *No* (absence of any incident). Below is additional information on specific types (or forms) of violence or aggression.

*Verbal aggression (prior and reporting year)*. These variables captured incidents involving shouting angrily, insulting others, cursing, and threatening others or themselves. They were reported with a *Yes* (presence) or a *N*o (absence). The presence of verbal aggression in the reporting year with self-harm behaviours was presented as dual harm related to verbal aggression.

*Physical aggression against objects (prior and reporting year)*. These variables captured incidents involving slamming doors, throwing objects, kicking furniture or walls, breaking objects, and setting fires. They were reported with a *Yes* (presence) or a *N*o (absence). The presence of this type of aggression and self-harming behaviours in the reporting year was operationalised as dual harm related to physical aggression against objects.

*Physical aggression against others (prior and reporting year)*. These variables captured incidents involving threatening gestures, kicking, pushing, and attacking others. They were reported with a *Yes* (presence) or a *N*o (absence). The presence of self-harming behaviours and this type of aggression was operationalised as dual harm related to physical aggression against others.

*Inappropriate sexual behaviour (prior and reporting year)*. These variables captured any incidents involving making sexually inappropriate or suggestive statements, exposing self to others, voyeuristic behaviours, sexually touching others non-consensually, and coercive sexual activities. They were reported with a *Yes* (presence) or a *N*o (absence). Similar to other types of dual harm, the presence of inappropriate sexual behaviour(s) and self-harming behaviours in the reporting year was operationalised as dual harm related to inappropriate sexual behaviours.

*Dual harm*. The outcome variable, dual harm, was an operationalised term based on self-harming behaviours and violence towards others/objects within the reporting year. However, a history of dual harm was considered in individuals who had been involved in both self-harming behaviours and violence towards others/objects before the reporting year. Another variable was also investigated: the number of different types of dual harm within the different periods (during the reporting year or before the reporting year).

#### Sociodemographic characteristics

These included age in years, gender (male, female, and others), and level of education (divided into up to grade 8, between grade 9 and 13, and post-secondary education).

#### Clinical characteristics

This included a history of substance misuse based on court and hospital records/reports before becoming a forensic patient), a history of self-harm, a psychiatric diagnosis, and the presence of a comorbid psychiatric diagnosis.

### Statistical analysis

The statistical software for data science (STATA), version 17, was used for analysis. Descriptive statistics were presented using frequency and percentages for categorical variables, whereas mean and standard deviation were used for continuous variables. The relationship of dual harm with sociodemographic and clinical characteristics was determined using the chi-square test or t-test depending on the variable (i.e., chi-square for categorical variables and t-test for continuous variable [age]). We also conducted a subgroup inferential analysis to understand the relationship of dual harm with clinical-demographic variables based on specific types of violent or aggressive incidents. Logistic regression analysis was used to determine the sociodemographic and clinical characteristics independently associated with dual harm, based on the backward stepwise method with all variables considered.

The relationship between self-harming behaviours in the past 12 months and violence was determined using the chi-square test. Logistic regression was used to determine the factors associated with past year self-harming behaviours. The final model controlled for sociodemographic and clinical characteristics.

During the analysis, a p-value of less than 0.05 was considered statistically significant, and we reported the corresponding 95% confidence interval. For multi-logistic regression, we used the Bonferroni Correction to address multiple testing, adjusting the significance level by dividing it by the number of tests performed.

## Results

### Characteristics of study patients

A total of 1240 forensic patients under the Ontario review board during 2014 and 2015 were included in the current study. The mean age of patients was 42.54 (±13.32) years, and the majority were male (85.73%). The largest proportion of individuals was managed for a psychotic-related disorder (81.61%). Approximately 15.22% had a history of self-harming behaviour, 14.20% had a prior dual harm history, and only 28.78% had never used any substance of addiction (See **Table 1**).

**Table 1:** Sociodemographic and clinical characteristics of patients under the Ontario Review Board in 2014 and 2015.

Variables			
**Demographic characteristics**			
**Age (mean ±standard deviation) 42.54 ±13.32 years**			
Description of variables and sub-groups		Frequency, n	percentage, %
Gender	Male	1063	85.73
	Female	173	13.95
	Others	4	0.32
Highest level of education	Up to grade 8	125	10.92
	Between grade 9 and 13	618	53.97
	Post secondary education	402	35.11
**Clinical characteristics**			
History of substance misuse	No	348	28.78
	Yes	861	71.22
History of self-harming	No	1014	84.78
	Yes	182	15.22
Psychiatry diagnosis	Psychosis	1012	81.61
	Mood disorders	83	6.69
	Neurodevelopmental disorder	33	2.66
	Personality disorder	54	4.35
	Others	58	4.68
Comorbid psychiatric illness	No	311	25.08
	Yes	929	74.92
History of dual harm before the reporting year	No	1039	85.80
	Yes	172	14.20

### Prevalence of dual harm and subtypes of dual harm

A total of 43 out of 1240 (3.55%) individuals engaged in dual harm within the past 12 months in reference to the reporting period. The majority of these cases involved dual harm related to verbal aggression (3.15%), followed by aggression towards objects (2.97%), and aggression towards others (2.73%). Sexually inappropriate behaviors related to dual harm were rarely reported within the past 12 months (1.32%). A total of 12 individuals experienced all forms of dual harm within the past 12 months. (See **Table 2**).

**Table 2:** Disaggregation of past 12-month dual harm based on the experience of one or multiple types of violence.

Forms of dual harm based on experience of one or multiple types of violence	n (%)
Dual harm related to verbal aggression (A)	39 (3.15)
Dual harm realed to aggression towards objects (B)	36 (2.97)
Dual harm related to aggression towards people (C)	33 (2.73)
Dual harm ralated to sexual violence (D)	16 (1.32)
A only	2 (0)
B only	1 (0)
C only	0 (0)
D only	1 (0)
Both A and B	4 (0)
Both A and C	2 (0)
Both A and D	1 (0)
Both B and C	2 (0)
Both B and D	0 (0)
Both C and D	0 (0)
A and B and C	16 (1)
A and B and D	1 (0)
A and C and D	1 (0)
B and C and D	0 (0)
A and B and C and D	12 (1)
None of harm subtypes	1197 (97)

The lifetime prevalence of dual harm (i.e., past year and/or history of dual harm prior to the reporting year) in the current population was 17.75% (215/1211). Of the 172 individuals with a history of dual harm prior to the reporting year, 28 (16.28%) of them were involved in dual harm within the reporting year.

### Relationship of dual harm with sociodemographic and clinical factors

Engaging in dual harm was most prevalent among individuals who had attained lesser education compared to those with higher levels of education, and this difference was statistically significant (all p-values less than 0.05). Dual harm occurred at a higher rate among individuals with a prior history of dual harm before the reporting year, and this relationship was maintained across the various forms of dual harm based on subtypes of violence (p-value <0.001). Our findings indicate a statistically significant difference in the relationship of dual harm with psychiatric diagnoses, with individuals with neurodevelopmental disorders engaging in the highest prevalence of dual harm. For details, see **Table 3**.

**Table 3:** Sociodemographic and clinical characteristics of patients.

Variables		Overall Dual harm			Verbal aggression related dual harm			Physical aggression against objects related dual harm			Physical aggression towards others related dual haem			Inappropriate sexual behavior related self-harm		
		No (%)	Yes (%)	t/χ^2^ (p-value)	No (%)	Yes (%)	t/χ^2^ (p-value)	No (%)	Yes (%)	t/χ^2^ (p-value)	No (%)	Yes (%)	t/χ^2^ (p-value)	No (%)	Yes (%)	t/χ^2^ (p-value)
		1168 (96.4)	43 (3.55)		1201 (96.85)	39 (3.15)		1175 (97.03)	36 (2.97)		1178 (97.27)	33 (2.73)		1195 (98.68)	16 (1.32)	
**Demographic characteristics**																
Age (mean, standard deviation)		42.69 (13.40)	38.79 (12.17)	1.88 (0.061)	42.65 (13.41)	39.31 (11.95)	1.54 (0.124)	42.68 (13.40)	38.14 (11.47)	**2.01 (0.045)**	42.66 (13.40)	38.54 (11.49)	1.74 (0.081)	42.55 (13.41)	42.69 (10.63)	-0.04 (0.966)
Gender	Male	1003 (96.43)	37 (3.56)	0.111 (0.946)	1006 (96.73)	34 (3.27)	0.14 (0.932)	1008 (96.92)	32 (3.08)	0.33 (0.846)	1012 (97.31)	28 (2.69)	0.13 (0.938)	1025 (98.56)	15 (1.44)	0.84 (0.658)
	Female	162 (96.43)	6 (3.57)		163 (97.02)	5 (2.98)		164 (97.62)	4 (2.38)		163 (97.02)	5 (2.98)		167 (99.40)	1 (0.60)	
	Others	3 (100)	0		3 (100)	0		3 (100)	0		3 (100)	0		3 (100)	0	
Highest level of education	Up to grade 8	116 (92.80)	9 (7.20)	**9.31 (0.010)**	116 (92.80)	9 (7.20)	**11.98 (0.003)**	117 (93.60)	8 (6.40)	**9.06 (0.011)**	117 (93.60)	8 (6.40)	**11.52 (0.003)**	117 (93.60)	8 (6.40)	**11.52 (0.003)**
	Between grade 9 and 13	581 (96.83)	19 (3.17)		581 (96.83)	19 (3.17)		585 (97.50)	15 (2.50)		584 (97.33)	16 (2.67)		584 (97.33)	16 (2.67)	
	Post secondary education	390 (98.24)	7 (1.76)		392 (98.74)	5 (1.26)		391 (98.49)	6 (1.51)		393 (98.99)	4 (1.01)		393 (98.99)	4 (1.01)	
**Clinical characteristics**																
History of substance misuse	No	325 (95.31)	16 (4.69)	2.15 (0.142)	327 (95.89)	14 (4.11)	1.51 (0.219)	326 (95.60)	15 (4.40)	3.46 (0.063)	327 (95.89)	14 (4.11)	3.57 (0.059)	338 (99.12)	3 (0.88)	0.58 (0.448)
	Yes	817 (97.03)	25 (2.97)		819 (97.27)	23 (2.73)		822 (97.62)	20 (2.38)		824 (97.86)	18 (2.14)		830 (98.57)	12 (1.43)	
Primary psychiatric diagnosis	Psychosis	958 (97.06)	29 (2.94)	**19.68 (0.001)**	961 (97.37)	26 (2.63)	**16.20 (0.003)**	965 (97.77)	22 (2.23)	**26.66 (<0.001)**	968 (98.07)	19 (1.93)	**30.76 (<0.001)**	977 (98.99)	10 (1.01)	**10.91 (0.028)**
	Mood disorders	79 (97.53)	2 (2.47)		79 (97.53)	2 (2.47)		79 (97.53)	2 (2.47)		79 (97.53)	2 (2.47)		80 (98.77)	1 (1.23)	
	Neurodevelopmental disorder	28 (84.85)	5 (15.15)		29 (87.88)	4 (12.12)		28 (84.85)	5 (15.15)		28 (84.85)	5 (15.15)		33 (100)	0	
	Personality disorder	48 (90.57)	5 (9.43)		48 (90.49)	5 (9.43)		48 (90.57)	5 (9.43)		48 (90.57)	5 (9.43)		50 (94.34)	3 (5.66)	
	Others	55 (96.49)	2 (3.51)		55 (96.49)	2 (3.51)		55 (96.49)	2 (3.51)		55 (96.49)	2 (3.51)		55 (96.49)	2 (3.51)	
Comorbid psychiatric illness	No	295 (98.01)	6 (1.99)	2.84 (0.092)	296 (98.34)	5 (1.66)	3.12 (0.077)	296 (98.34)	5 (1.66)	2.39 (0.122)	296 (98.34)	5 (1.66)	1.71 (0.191)	301 (100)	0	**5.36 (0.021)**
	Yes	873 (95.93)	37 (4.07)		876 (96.26)	34 (3.74)		879 (96.59)	31 (3.41)		882 (96.92)	28 (3.08)		894 (98.24)	16 (1.76)	
History of dual harm before the reporting year	No	1024 (98.56)	15 (1.44)	**94.84 (<0.001)**	1027 (98.85)	12 (1.15)	**100.13 (<0.001)**	1028 (98.94)	11 (1.06)	**92.81 (<0.001)**	1031 (99.23)	8 (0.77)	**105.48 (<0.001)**	1031 (99.23)	8 (0.77)	**105.48 (<0.001)**
	Yes	144 (83.72)	28 (16.28)		145 (84.30)	27 (15.70)		147 (85.47)	25 (14.53)		147 (85.47)	25 (14.53)		147 (85.47)	25 (14.53)	

Individuals involved in dual harm related to aggression against objects were significantly younger compared to those who were not engaged in dual harm related to aggression against objects (mean age in years: 38.14±11.47 vs. 42.68±13.40, t = 2.01, *p*-value = 0.045). No statistical difference in age was observed when we considered the presence of dual harm or from all sub-analysis of dual harm based on different types of violence. Having comorbid psychiatric conditions was associated with dual harm related to inappropriate sexual behavior (0 vs. 1.76, χ^2^ = 5.36, *p*-value = 0.021). This statistical difference based on psychiatric comorbidity was not present with dual harm or any subtypes of dual harm based on the different nature of violent or aggressive incidents (**Table 3)**.

### Clinical and sociodemographic factors associated with subtypes of dual harm based on various types of violent or aggressive incidents

Consistently across the various types of dual harm, the likelihood of dual harm significantly increased with having a previous history of dual harm before the reporting year, an aspect that remained significant after correction for multiple testing. Also, with the exception of dual harm related to inappropriate sexual behavior, having a diagnosis of a personality disorder was not associated with subtypes of dual harm. However, a diagnosis of neurodevelopmental disorder was associated with an increase in the likelihood of all forms of dual harm. However, this only remained significant after correction for multiple testing with dual harm involving physical harm to objects and others. With respect to sociodemographic characteristics, post-secondary educational level was associated with a reduction in the likelihood of engaging in dual harm, except for dual harm related to inappropriate sexual behaviors. However, based on the Bonferroni Correction, post-secondary education was linked to a reduced likelihood of verbal aggression-related dual harm. Finally, increasing age was associated with a reduction in the likelihood of engaging in dual harm in general, and dual harm related to verbal aggression and physical aggression towards objects (See **Table 4)**.

**Table 4:** Sociodemographic and clinical characteristics associated with dual harm.

Variable		Verbal aggression related dual harm		Physical aggression against objects related dual harm		Physical aggression towards others related dual haem		Inappropriate sexual behavior related self-harm		Overall dual harm	
		aOR (95% CI)	*p*-value	aOR (95% CI)	*p*-value	aOR (95% CI)	*p*-value	aOR (95% CI)	*p*-value	aOR (95% CI)	*p*-value
Age		0.96 (0.93–1.00)	**0.036**	0.96 (0.93–1.00)	**0.040**	0.96 (0.93–1.01)	0.066	0.99 (0.95–1.04)	0.735	0.97 (0.93–1.00)	**0.043**
Gender	Male	1 (reference)		1 (reference)		1 (reference)		1 (reference)		1 (reference)	
	Female	0.70 (0.23–2.07)	0.515	0.58 (0.18–1.92)	0.376	0.82 (0.27–2.55)	0.737	0.38 (0.04–3.26)	0.376	0.89 (0.33–2.41)	0.816
	Others	1 (omitted)		1 (omitted)		1 (omitted)		1 (omitted)		1 (omitted)	
Highest level of education	Up to grade 8	1 (reference)		1 (reference)		1 (reference)		1 (reference)		1 (reference)	
	Between grade 9 and 13	0.43 (0.16–1.33)	0.088	0.39 (0.14–1.10)	0.075	0.44 (0.15–1.27)	0.131	1.03 (0.22–4.91)	0.965	0.43 (0.17–1.12)	0.084
	Post secondary education	0.18 (0.05–0.62)	0.007*	0.27 (0.08–0.93)	**0.038**	0.17 (0.04–0.67)	**0.011**	1.08 (0.19–6.11)	0.932	0.26 (0.08–0.82)	**0.022**
History of substance misuse	No	1 (reference)		1 (reference)		1 (reference)		1 (reference)		1 (reference)	
	Yes	0.58 (0.21–1.56)	0.279	0.55 (0.20–1.55)	0.263	0.53 (0.18–1.55)	0.246	1.64 (0.30–8.89)	0.563	0.66 (0.25–1.71)	0.394
Primary psychiatric diagnosis	Psychosis	1 (reference)		1 (reference)		1 (reference)		1 (reference)		1 (reference)	
	Mood disorders	2.80 (0.58–13.55)	0.199	3.17 (0.65–15.51)	0.154	4.16 (0.80–21.52)	0.089	2.62 (0.27–25.08)	0.404	2.09 (0.44–9.84)	0.351
	Neurodevelopmental disorder	6.54 (1.53–27.97)	**0.011**	8.03 (1.89–34.16)	0.005*	8.54 (1.85–39.42)	0.006*	1 (omitted)		5.82 (1.44–23.46)	**0.013**
	Personality disorder	1.28 (0.21–7.75)	0.785	1.69 (0.27–10.35)	0.571	1.61 (0.25–10.38)	0.618	8.39 (1.22–57.83)	**0.031**	1.33 (0.23–7.74)	0.753
	Others	1.55 (0.28–8.41)	0.614	1.98 (0.36–11.03)	0.434	2.04 (0.36–11.60)	0.423	5.09 (0.85–30.50)	0.075	1.48 (0.28–7.74)	0.644
Comorbid psychiatric illness	No	1 (reference)		1 (reference)		1 (reference)		1 (reference)		1 (reference)	
	Yes	1.29 (0.37–4.50)	0.686	1.66 (0.42–6.59)	0.473	1.46 (0.36–5.99)	0.599	1 (omitted)		1.48 (0.44–4.97)	0.530
Past history of dual harm	No	1 (reference)		1 (reference)		1 (reference)		1 (reference)		1 (reference)	
	Yes	17.32 (7.49–40.03)	<0.001*	16.01 (6.59–38.90	<0.001*	22.81 (8.65–60.18)	<0.001*	17.69 (5.13–60.93)	<0.001*	13.17 (6.05–28.67)	<0.001*

*Significant after correction for multiple testing based on Bonferroni Correction

**Bold number—**p-value less than 0.05

### Relationship between past 12-month self-harming behaviour and different forms of violence

Of the total sample, 29 individuals had no information concerning past 12-month self-harming behaviours. Approximately 3.55% of the patients were involved in self-harming behaviours in the reporting year. We found statistically significant differences between self-harming behaviour and all forms of violence (past and the reporting year) except previous verbal aggression. The largest difference was observed with previous 12-month engagement in aggression towards others and having engaged in self-harm in the previous 12 months. (26.28% vs. 0.56%, χ2 = 239.60, p-value <0.001). See **Table 5** for details.

**Table 5:** Relationship between past 12-month self-harming behaviors and different types of violence.

Variable	n (%)	Past 12-months self-harming behavior; n (%)	χ^2^ (p-value)
		43 (3.55)	
**Lifetime presence of violence prior to reporting year**			
**Any inpatient aggression or acting violently before the review period**			
No	433 (35.99)	4 (0.93)	**14.02 (0.001)**
Yes	744 (61.85)	36 (4.94)	
**Verbal aggression**			
No	415 (34.64)	10 (2.43)	2.07 (0.150)
Yes	783 (65.36)	31 (4.05)	
**Physical aggression against objects**			
No	825 (68.92)	15 (1.85)	**20.71 (<0.001)**
Yes	372 (31.08)	26 (7.10)	
**Physical aggression against others**			
No	581 (48.54)	7 (1.22)	**16.99 (<0.001)**
Yes	616 (51.46)	34 (5.63)	
**Inappropriate sexual behaviours**			
No	847 (70.76)	16 (1.93)	**20.51 (<0.001)**
Yes	350 (29.24)	25 (7.25)	
**Past 12-month violence incidences**			
**Verbal aggression**			
No	871 (71.98)	4 (0.46)	**87.14 (<0.001)**
Yes	339 (11.33)	39 (11.54)	
**Physical aggression against objects**			
No	1072 (88.67)	6 (0.56)	**239.60 (<0.001)**
Yes	137 (11.33)	36 (26.28)	
**Physical aggression against others**			
No	1036 (85.55)	10 (0.97)	**139.94 (<0.001)**
Yes	175 (14.45)	33 (18.86)	
**Inappropriate sexual behaviors**			
No	1086 (89.68)	27 (2.49)	**34.82 (<0.001)**
Yes	125 (10.32)	16 (12.80)	

### Strength of the relationship between self-harming behaviors and violence after controlling for clinical-demographic confounders

Following bivariate analysis, the factors associated with self-harming behaviour were entered into a multivariate logistic regression model, controlling for social, demographic, and clinical characteristics (See **Table 6**). The factors associated with self-harming behaviour included recent involvement in aggression towards others, objects, verbal, and inappropriate sexual behaviour towards others. The odds of self-harm were highest with aggression to objects (this was also significant after correction for multiple testing), followed by verbal aggression, and aggression to humans (i.e., inappropriate sexual behaviours and aggression to humans that include both verbal and physical) had the least odds.

**Table 6:** Violence history associated with past 12-month self-harming behaviors among forensic psychiatry patients.

Variable	Bivariate analysis		Multivariate analysis	
	Crude odds ratio (95% confidence Interval)	p-value	Adjusted odds ratio (95% confidence Interval)	p-value
**Lifetime presence violence prior to reporting year**				
**Any inpatient aggression or acting violently before the review period**				
No	1 (reference)		1 (reference)	
Yes	5.51 (1.95–15.58)	**0.001**	0.51 (0.07–3.55)	0.500
Outpatient (no previous institutionalization)	1 (omitted)		1 (omitted)	
**Verbal aggression**				
No	1 (reference)			
Yes	1.69 (0.82–3.48)	0.154		
**Physical aggression against objects**				
No	1 (reference)		1 (reference)	
Yes	4.06 (2.12–7.76)	**<0.001**	0.44 (0.12–1.62)	0.218
**Physical aggression against others**				
No	1 (reference)		1 (reference)	
Yes	4.82 (2.12–10.97)	**<0.001**	0.72 (0.12–4.24)	0.720
**Inappropriate sexual behaviours**				
No	1 (reference)		1 (reference)	
Yes	3.98 (2.10–7.55)	**<0.001**	0.82 (0.23–2.97)	0.764
**Past 12-month violence**				
**Verbal aggression**				
No	1 (reference)		1 (reference)	
Yes	28.27 (10.02–79.78)	**<0.001**	6.74 (1.16–39.07)	**0.033**
**Physical aggression against objects**				
No	1 (reference)		1 (reference)	
Yes	63.33 (26.06–153.90)	**<0.001**	11.75 (3.26–42.35)	<0.001*
**Physical aggression against others**				
No	1 (reference)		1 (reference)	
Yes	23.84 (11.50–49.42)	**<0.001**	4.55 (1.39–14.90)	**0.012**
**Inappropriate sexual behaviors**				
No	1 (reference)		1 (reference)	
Yes	5.76 (3.01–11.02)	**<0.001**	3.41 (1.05–11.10)	**0.042**
**Model statistics**				
Pseudo r2			0.55	<0.001
Goodness of fit (x2)			858.92	0.9110
Sensitivity			54.84%	
Specificity			99.51%	
PPV			77.27%	
NPV			98.64%	
Correctly classifies			98.19%	
VIF			Mean = 1.37 (all below 2)	

Controlled for sociodemographic and clinical factors.

*Significant after correction for multiple testing based on Bonferroni Correction

**Bold number—**p-value less than 0.05

## Discussion

### Prevalence of dual harm

Approximately 3.55% of forensic patients in Ontario engaged in dual harm in the reporting year. This is a clinically significant burden among individuals due to the severe impacts of the potential outcomes of dual harm, such as death, physical deformities to others, and destruction of property. This prevalence of reported dual harm was lower than 19% among patients admitted to a forensic hospital in Sweden between 1992 and 1994 [10]. The higher prevalence from this Swedish study may be attributed to various reasons, including (i) the study had a small sample size (n = 58), limiting the ability to determine the burden of dual harm accurately, (ii) dual harm not being based on violent criminality and lifetime history of self-harm—these aspects do not reflect patients’ recent status while in the forensic system. Interestingly, this Swedish study by Stålenheim [10] excluded individuals with psychosis, who are known to have higher incidences of engaging in dual harm [27].

All in all, few studies have explored dual harm among individuals in the criminal justice system, especially forensic patients, and it is noteworthy that most studies have concentrated on individuals in prisons [3]. Even among studies done in correctional institutions, the focus has been mainly on dual harm related to physical and verbal aggression. Dual harm related to inappropriate sexual behaviors has been relatively neglected or understudied.

Dual harm related to verbal aggression was more prevalent than other forms of dual harm. This may be because it is less risky to be involved in verbal aggression than other forms of aggression. Moreover, it is more socially acceptable, and despite involving no physical contact, it may be more effective in achieving certain goals such as influencing, persuading, or intimidating [28].

### Clinical and sociodemographic factors associated with dual harm

In the present study, having a diagnosis of a neurodevelopmental disorder was associated with an increased likelihood of exhibiting dual harm during the reporting year. This was consistent with almost all types of dual harm except dual harm related to inappropriate sexual behaviors. This finding appears to be consistent with previous observations indicating that individuals with neurodevelopmental disorders often experience difficulties with emotional dysregulation, characterised by anger, sadness, or frustration [29, 30]. Moreover, they may have difficulty expressing their emotions appropriately in stressful situations [29]. As a result of these emotional dysregulations, they can resort to maladaptive or impulsive behaviors such as self-harm and aggression (dual harm) to cope with their situations [7]. Despite this possible relationship, a previous study by Laporte et al. [31] found no association between deliberate self-harm among violent offenders (dual harm) and neurodevelopmental disorders in a Swedish study.

The current study results show that dual harm related to past years of inappropriate sexual behaviours was associated with personality disorders. Despite not having conducted a deeper analysis on the types of personality disorders or the use of psychometric tools to identify certain personality traits, some certain types of personality disorders (including borderline and antisocial personality disorder) have a strong association with dual harm, according to prior research [7, 32, 33]. Some personality traits and disorders have also been strongly associated with inappropriate sexual behaviors [34], hence supporting the relationship observed in the present study.

As reported in previous studies [7, 12], it is unsurprising that a history of dual harm was the strongest predictor of a recent incident of dual harm in the current study. This finding may be related to individuals using the same strategies to cope with challenging behaviors [7]. Regarding demographic factors, attaining a higher education level was associated with a reduced likelihood of dual harm. It is plausible to attribute this to a greater likelihood of individuals learning and adopting better coping strategies as they go through school.

### Relationship between self-harming behaviours and violence

Among forensic patients under the ORB, violence is associated with self-harming behaviors, a finding similar to other studies among forensic patients [35, 36]. In the current study, the history of violence associated with self-harming behaviors over the past 12 months included aggression towards others, objects, verbal aggression, and inappropriate sexual behavior. Self-harming behaviors were significantly more associated with aggression toward objects than aggression toward humans (verbal, physical, or sexual). While self-harm was most strongly related to aggression towards objects, it was also linked to other forms of aggression. As harming objects may result in fewer consequences, individuals may find this outlet a best-case scenario to express their frustration without harming another individual. Many individuals in the forensic system in Ontario were admitted following the incidents and consequences of aggression to others (humans) [i.e., 69.9%]. Consequently, they may have learned to avoid acting aggressively toward others by displacing their aggression toward objects [26].

In addition, some individuals prefer to be aggressive towards objects because they will not retaliate or resist like humans. Also, aggression towards objects may be a form of self-punishment or self-blame for perceived failures, mistakes, or shortcomings [37]. Some people may feel guilty or ashamed of their self-harming behaviours and project their self-criticism onto objects representing themselves or their problem(s). Objects can be used to signify individuals’ problems and can harm them every time they are stressed. Harming these objects can be a substitute for self-harming behaviours, albeit more studies are needed to unpack this relationship.

### Study limitations

The present study had a few limitations, including its cross-sectional study design. Hence, causality could not be inferred. We recommend future studies track the events prospectively and explore for association based on the number of incidents. Secondly, the study was among individuals in a controlled environment who are strictly monitored and may be restrained from engaging in dual harm behaviors. This may lead to a lower burden of the incidents or prevalence of dual harm, and this may affect some subtypes of dual harm compared to others. For example, engaging in inappropriate sexual activities is likely to be less prevalent in a controlled environment. Hence, the lower odds compared to other forms of violence. The present study did not look at the association between the severity of the violence and self-harm incidents or the number of occurrences (s) that could have affected the dual harm relationship and its associated factors. Taken together, we recommend that future studies look into the qualitative description of different aspects or scenarios of dual harm to understand the phenomena better. Lastly, we acknowledge that our study relied on records/reports to obtain mental health history and other relevant data, which may introduce a degree of unreliability carried forward from the original data capture. For future studies, we recommend prospective studies to overcome this limitation.

### Study implications and recommendations

This study provides information on dual harm among patients in forensic psychiatric systems, which constitutes a dissection into clinically significant struggles for patients. Our study findings represent an extension of existing literature, reporting the burden and associated factors of dual harm in the forensic population. We recommend that clinicians, correctional officers, and law enforcement agencies be cognizant of this and screen for dual harm. This is particularly important because the consequences of dual harm can be fatal, and many individuals who harm others or objects are at a high risk of harming themselves and vice versa. We also encouraged targeted intervention and supportive monitoring for individuals with a history of dual harm, neurodevelopmental disorder, and harm to self or others. Such intervention should include active follow-up based on what is known about vulnerability or the odds of engaging in future dual harm. To ensure that preventive interventions and mitigating measures are effectively implemented, policymakers and other relevant stakeholders must actively engage to provide adequate funding and allocation of resources. Trial studies to test the effectiveness of interventions and better understand modifiable risks of dual harm are indicated.

## Conclusion

Dual harm is prevalent among forensic patients in Ontario, with self-harm being most common among those exhibiting various forms of aggression, particularly towards objects. To mitigate dual harm and its associated complications, it is crucial to implement strategies that manage the risk of self-harm among aggressive patients. This is especially important for individuals with neurodevelopmental disorders, who may be more vulnerable to dual harm. In addition, strategies to improve education are encouraged due to its protective nature towards some forms of dual harm. Comprehensive interventions and supportive monitoring should be prioritized to address these risks effectively.
